# Monitoring microplastics in coastal waters of a biosphere reserve: a case study in Menorca (Spain)

**DOI:** 10.1007/s11356-023-31061-y

**Published:** 2023-11-30

**Authors:** Carme Alomar, Beatriz Rios-Fuster, Maria Elena Cefalì, Valentina Fagiano, Salud Deudero

**Affiliations:** 1 Instituto Español de Oceanografía, Centro Oceanográfico de Baleares (IEO, CSIC), Muelle de Poniente S/N, 07015 Palma, Mallorca Spain; 2 Estació d’Investigació Jaume Ferrer, La Mola, Centro Oceanogràfico de Baleares, IEO-CSIC, PO Box 502, 07701 Mao, Menorca Spain

**Keywords:** Plastic pollution, Marine ecosystems, Human impacts, Marine Protected Areas, Waste management

## Abstract

This study provides with evidence of the presence of sea surface microplastics in a UNESCO marine biosphere reserve: the island of Menorca in the north-western Mediterranean Sea. From a total of 90 samples, in 100% of the samples, microplastics were observed with a mean value of 0.18 ± 0.01 items/m^2^. According to data, no significant differences were observed for sampling period with very similar values between 2021 (0.17 ± 0.02 items/m^2^) and 2022 (0.18 ± 0.02 items/m^2^). However, significant differences were observed regarding sampling area (both site and locality) suggesting that sea surface plastics in the study area might be more dependent of the spatial scale rather than on the temporal scale. Fibre type microplastics predominated over fragments, films, pellets, and foams, but in the commercial *Port de Maó*, almost 50% of the identified items were foams which could be related to the transportation of packed goods to this port. Results from the model applied to study the relation between waste management indicators and microplastic abundance indicate that when considering all marine litter categories, the explanatory variables are plastic waste generated by residents population (tonnes/year/km^2^) and waste collection rate (%), whereas if only plastics are considered, the indicator regarding waste per capita (kg/hab/year) is also included. Data in this study is obtained through a harmonized protocol which can be used to define baseline and threshold values to evaluate good environmental status regarding descriptor 10 of the Marine Strategy Framework Directive.

## Introduction

In the last decade, science has given more than enough evidence that plastic pollution is widely present and spread in all sea environments from sea surface to seafloor, including the water column and coast with global estimated values ranging from 53,500 to 3,546,700 tonnes in the Mediterranean Sea (Boucher and Bilard 2020). Given the nature of the Mediterranean Sea, a semi-enclosed basin with limited surface water exchange with the Atlantic Ocean (Schmidt et al. 2018) along with the existing intense human coastal pressures (tourism, fishing, maritime traffic, industry, and dense coastal urbanization), this area is one of the most worldwide impacted region regarding plastic pollution (Macias et al. 2019). A recent study has documented microplastic (MP) abundance ranging from 5576 to 379,965 items/km^2^ along the sea surface of north-western Mediterranean harbours possibly linked to the fact that these areas hold industrial and transportation activities which are important entering points of plastic pollution to the ocean (Tesán Onrubia et al. 2021). Not only anthropogenic areas are exposed to plastic pollution but some studies indicate that Marine Protected Areas (MPAs) or areas with some type of protection status can be even more exposed to plastic pollution than anthropogenic areas (Compa et al. 2022a, b). Additionally, similar sea surface plastic abundances have been quantified in protected areas, with no local land-based contamination sources, and high anthropized areas in the western Mediterranean Sea (Fagiano et al. 2022). Moreover, plastics quantified in the protected areas were made up of small items, mainly fragments (Fagiano et al. 2022), which could be indicating the transportation and fragmentation of larger plastic items into smaller particles in the marine environment. Marine protected areas are frequently made up of small islands and islets which are generally exposed to meteorological factors that might enhance plastic accumulation and retention (Compa et al. 2022a, b). In this sense, there is scientific evidence that microplastic abundances deposited in sediments are higher in MPAs than in areas exposed to human pressures (Alomar et al. 2016; Cohen-Sánchez et al. 2022), and its distribution can also be affected by the characteristics of the seafloor (Rios-Fuster et al. 2023). Due to currents and hydrodynamics, plastics can be transported across seas and oceans and transboundary pollution in MPAs is a reality (Compa et al. 2020; Hatzonikolakis et al. 2022). In this sense, there is scientific evidence that in the Mediterranean basin, all countries have at least one national MPA with over 55% of macroplastics originating from sources beyond their borders and predicted concentrations of microplastics and macroplastics are high in national MPAs and Natura 2000 sites (Hatzonikolakis et al. 2022).

The Balearic Islands, located in the western Mediterranean Sea, are vulnerable to plastic pollution, especially during the high touristic season, in summer, in which its population increases drastically and this fact is reflected in microplastic abundances quantified in marine ecosystems of this area (Compa et al. 2022a, b). In this sense, sea surface water samples collected during August around coastal areas contained almost two-fold as much plastic than samples obtained during July and September and the number of items decreased significantly with distance from the coastline (Compa et al. 2020). Plastic sources can be sea-based (20%) or land-based (80%) and backtracking simulations from Compa et al. (2020) indicated that in the Balearic Islands, marine litter was mainly locally sourced. However, these islands are situated in the middle of the Algerian and Balearic sub-basins (Acosta et al. 2004),under the influence of the Northern and Algerian Currents, which can potentially be transferring plastics from more distant areas to these islands (Alomar 2020). Consequently, these islands are exposed to land and sea based plastic pollution from local sources but also from distant sources. Amongst the Balearic Islands, Menorca, situated in the northern part of the archipelago, was declared a biosphere reserve by UNESCO in 1993 and the marine environment was included as part of this reserve in 2019. Floating plastics have already been quantified in the Menorca Channel, between the island of Mallorca and Menorca (Ruiz-Orejón et al. 2019), and results suggested that plastic litter was persistent around the sampling area and throughout the sampling period with microplastics and rigid particles being the most frequent observed categories (Ruiz-Orejón et al. 2019).

In order to understand distribution patterns and fate of microplastics in the marine environment, in situ data in addition to information regarding human activities which can be influencing the amount of plastics entering the marine should be considered. In this way, general waste management, recycling, wastewater and run-off water management, plastic consumption patterns, population density, value of plastic polymer, type of use, distance to shores and rivers, and hydrological patterns are all parameters which in one way or another are influencing plastic leakage to rivers and oceans (IUCN-EA-QUANTIS 2020).

The Research Station Jaume Ferrer in la Mola, Menorca, has the execution of monitoring programs in a sustained manner amongst its goals allowing to obtain the necessary indicators for the assessment and management of marine ecosystems around Menorca. Amongst these programs, plastic pollution monitoring aims to quantify and evaluate the presence of plastic pollution around coastal waters of Menorca at a micro spatial and temporal scale. Consequently, the main aim of this research is to quantify plastic loads around Menorca in an attempt to provide for the first time with a time series of this type of pollution in a marine biosphere reserve. As a second aim, this research attempts to asses which indicators related to waste management are more indicative of microplasti abundance along coastal waters of Menorca.

## Material and methods

### Study area

**Fig. 1:**
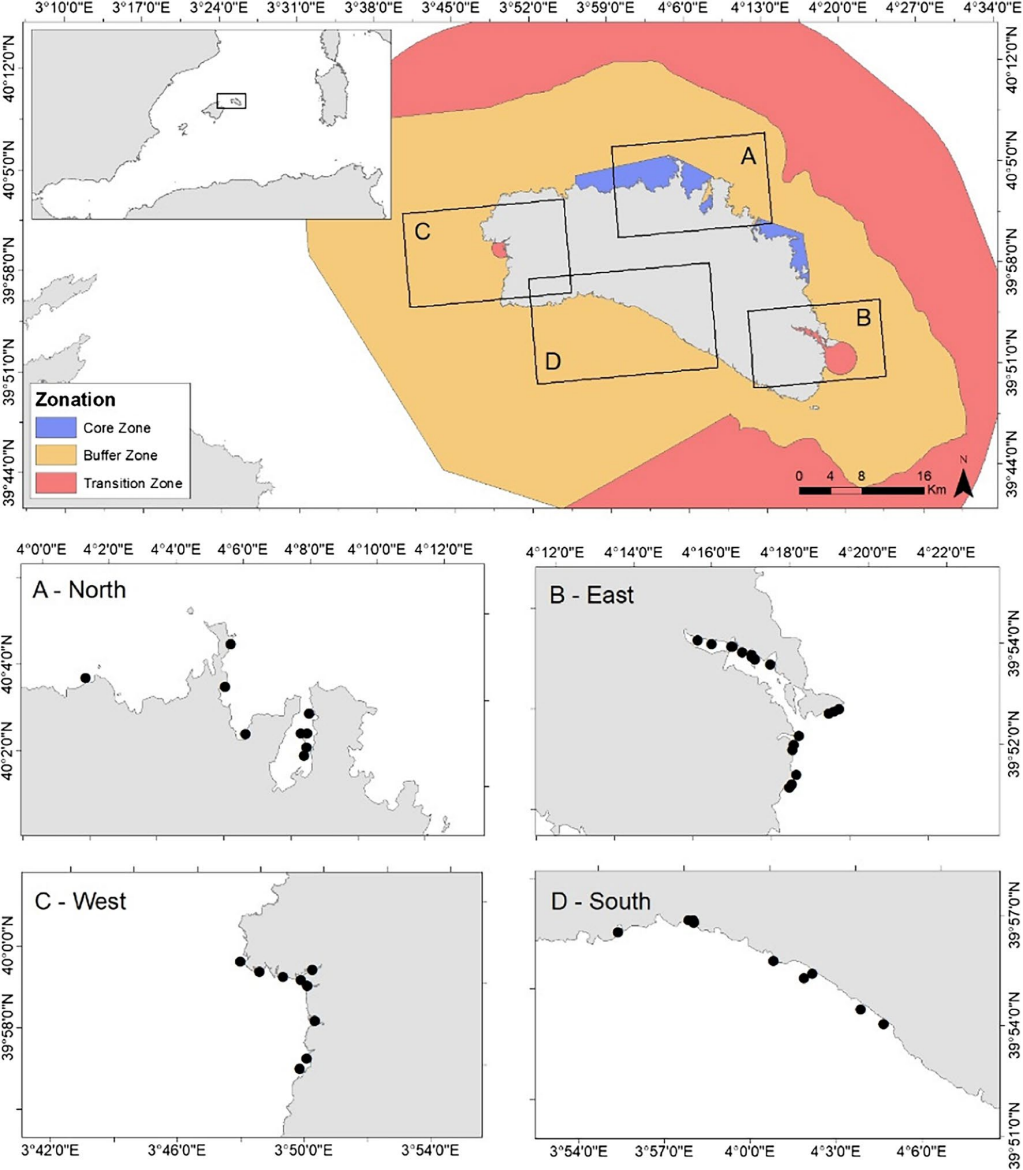
Map indicating sampling areas for the monitoring of plastics in coastal waters of Menorca Biosphere Reserve

Sea surface microplastics were sampled around Menorca (Fig. 1), a UNESCO biosphere reserve since 1993 and from 2019 this figure includes the marine environment. In order to quantify plastic pollution around Menorca, four macroareas, corresponding to the four cardinal points (east, south, west, and north) were sampled. Regarding the characteristics of the east site, two sampling sites were furthered considered: inside and outside the commercial *Port de Maó*. At each study area, three localities were sampled (Table 1) and samples were obtained in May, July, and October in 2021 and 2022.

**Table 1:** Sea surface microplastic (MP) abundances (mean ± SD) according to sampling site (east, north, south, and west) and locality around Menorca Biosphere Reserve. *n* indicates number of samples. In bold total number of samples according to sampling area, year and total sampling survey

	*n*	MPs/m^2^
East	**36**	**0.15 ± 0.01**
Commercial Port	6	0.18 ± 0.05
Illa del Rei	6	0.18 ± 0.04
La Mola	6	0.08 ± 0.02
Mussel Farm	8	0.21 ± 0.04
Rafalet	6	0.10 ± 0.01
Sant Esteve	6	0.13 ± 0.02
North	**18**	**0.22 ± 0.03**
Cala Tirant	1	0.45 ± n/a
Cavalleria	5	0.22 ± 0.03
Port Fornells	8	0.24 ± 0.04
Sanitja	4	0.13 ± 0.02
South	**18**	**0.13 ± 0.02**
Cala Galdana	7	0.13 ± 0.02
Macarella	4	0.18 ± 0.06
Santo Tomas	5	0.08 ± 0.03
Son Bou	2	0.17 ± 0.10
West	**18**	**0.24 ± 0.04**
Cala Blanca	6	0.16 ± 0.04
Cala en Blanes	6	0.28 ± 0.10
Port Ciutadella	6	0.29 ± 0.06
Total 2021	**45**	**0.17 ± 0.01**
Total 2022	**45**	**0.18 ± 0.02**
Total sampling survey	**90**	**0.18 ± 0.01**

### Field work

A total of 90 sea surface manta trawl samples were collected during 2021 and 2022. Sea surface was sampled by means of a manta trawl which consists of a metallic frame with a rectangular opening with dimensions of 40 × 70 cm and which is equipped with a cod length net of 2 m and a mesh size of 335 μm. The manta trawl was towed at an average speed between 1.5 and 3 nautical miles per hour during 10 min. The samples were transferred to a 500 ml bottle onboard and transported directly to the laboratory at the Research Station Jaume Ferrer in Menorca.

### Laboratory work

Once in the laboratory, all samples were filtered through a filtration ramp with a vacuum pump and using glass fibre filters. Filters were left to dry at room temperature in covered glass Petri dishes. Each filter was visually inspected under the stereomicroscope, and plastic items were quantified and classified according to shape (fragment, filament, pellet, fibre, and foam) and colours (black, blue, white, transparent, green, red, pink, yellow, orange, and brown) following standardized protocols (Galgani et al. 2013). The contamination of laboratory clothing, surfaces, and atmosphere, as well as other sources of MPs, was constantly monitored. All laboratory materials were cleaned with distilled water and 70% ethanol, and all the operators wore white cotton gowns. In addition, and in order to reduce potential airborne contamination, the Petri dishes were only opened when strictly necessary and fibre filters were inspected with the Petri dish lid closed.

### Fourier-transform infrared spectroscopy (FTIR)

Attenuated total reflection Fourier-transform infrared spectroscopy (ATR-FTIR) analysis was applied to a subset of the particles identified to determine the polymers composing these particles. For this sub-set sample, items were picked from the glass Petri dish and placed separately onto the ATR unit to be analysed with the platinum ATR of the Tensor 27 spectrometer (Bruker, Germany) at the University of the Balearic Islands. Smallest items (< 500 µm) were analysed directly on filters using an ATR crystal attached to a microscope (micro-FTIR).

The wavenumber range of 400–4000 cm^−1^ was used for measurements, and eight scans were performed per item. Each spectrum was compared with spectra from a customized polymer library integrating different databases (Löder et al. 2015; BASEMAN D1_2 FTIR reference database) and an in-house library generated with virgin and weathered reference polymers, including various natural and synthetic materials. Only samples with a quality hit index > 700 (max. 1000) were accepted as confirmed polymers. Spectra comparison was done with the Opus 6.5 software.

### Data analyses

In order to estimate the abundance of plastics per sampling locality (items/m^2^), once items were counted and categorized, the sea surface area trawled with the manta trawl was calculated according to the trawling distance and width of the manta net (Suaria et al. 2016). Plastic abundances (count) were expressed as mean value ± standard deviation (SD). To study significant differences in plastic abundance along coastal areas of Menorca according to year, month, sampling area and locality, a PERMANOVA analysis of four fixed factors: year, month, area, and locality was conducted. The variable items/m^2^ was transformed using the fourth root, and the resemblance matrix was built based on Euclidean distance.

Given that in the east two sampling sites were considered: inside and outside the *Port de Maó*, a separate analysis was conducted for this area. Specifically, a PERMANOVA analysis of three fixed factors: year, month, and locality was conducted. The variable items/m^2^ was also transformed using the fourth root, and the resemblance matrix was also built based on Euclidean distance. Statistical differences were established at *p* < 0.05, and these analyses were performed using the Primer V6 and the add-on package PERMANOVAþ (Anderson et al. 2008).

### Plastic waste management indicators

To identify the main waste management indicators related to marine litter and plastic abundance along the sea surface of coastal areas of Menorca, the following indicators were considered as continuous variables: plastic waste generated by resident population (tonnes/year/km^2^), waste generated on beach (tonnes/year), waste collection rate (%), leakage of macroplastics from resident population (tonnes/year/km^2^), leakage from tourist population (tonnes/year), total waste (kg), and waste per capita (kg/hab/year) as well as marine litter and plastic abundance. The best-fit model was selected corresponding to the lowest AIC value (Akaike Information Criterion) by applying the stepAIC function from the MASS package (Ripley et al. 2011). Normality was assessed upon inspection of the residuals of the model. These statistical analyses were performed in the R version 1.2.1335.

For all indicators except for total waste and waste per capita, median values for each of the sampling localities were obtained from the maps in the National Guidance for Plastic Pollution hotspoting and shaping action (IUCN-EA-QUANTIS 2020). Data was obtained in 2018, and each of the sampling localities was assigned to the nearest municipality for data recompilation. Data on waste collection rate was log transformed. For the total waste and waste per capita indicators, data was retrieved from the *Observatori Socioambiental de Menorca* (OBSAM) database.

## Results

### Abundances

**Fig. 2:**
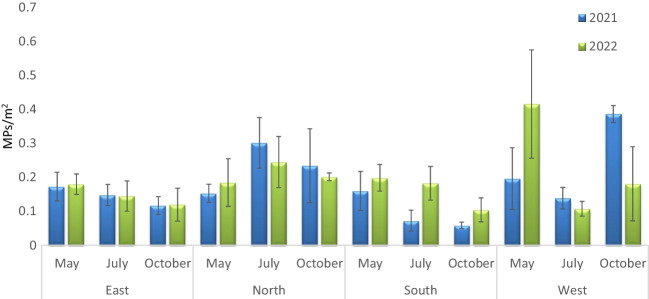
Sea surface microplastic (MP) abundances (mean ± SD) according to sampling area, month, and year around Menorca Biosphere Reserve

**Fig. 3:**
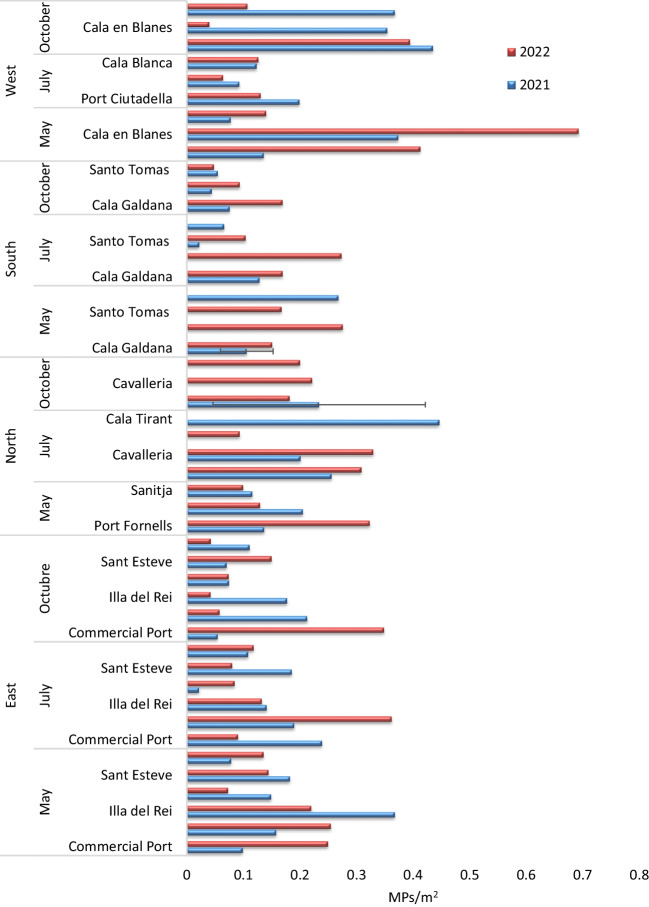
Sea surface microplastic (MP) abundances (mean ± SD) according to sampling locality, month, and year around Menorca Biosphere

A total of 90 sea surface manta trawl samples were collected during 2021 and 2022 with a mean microplastic abundance of 0.18 ± 0.01 items/m^2^ (Table 1). According to years, in 2021 a mean microplastic abundance of 0.17 ± 0.02 items/m^2^ was obtained and in 2022, a mean microplastic abundance of 0.18 ± 0.02 items/m^2^ was quantified but no significant differences were observed according to year (Table 2; *p* > 0.05; PERMANOVA). Regarding sampling months, no significant differences were also observed (Table 2; *p* > 0.05; PERMANOVA) and highest mean values, considering both study years, were given in May (0.20 ± 0.02 items/m^2^), while the same abundance of floating microplastics were observed in July and October (0.16 ± 0.02 items/m^2^). Considering years, in 2021 highest mean microplastic abundances were obtained during October (0.18 ± 0.04 items/m^2^) and lowest abundances in May (0.17 ± 0.02 items/m^2^) and these differences were not significant (Table 2; *p* > 0.05; PERMANOVA). On the other hand, in 2022 a reverse trend was observed as highest mean microplastic values were observed during May (0.23 ± 0.04 items/m^2^) and lowest values in October (0.15 ± 0.03 items/m^2^) (Table 2; *p* > 0.05; PERMANOVA) (Fig. 2). Regarding sampling areas and considering both years, significant differences were observed between areas (Table 2; *p* < 0.01; PERMANOVA) with highest mean microplastic values observed in the west (0.24 ± 0.04 items/m^2^) and lowest in the south (0.13 ± 0.02 items/m^2^) of Menorca. When considering sampling localities, significant differences were observed (Table 2; *p* < 0.05; PERMANOVA) with highest mean abundances observed in the west, *Port de Ciutadella* (0.29 ± 0.04 items/m^2^), excluding *Cala Tirant* since it was only sampled once, and lowest values observed both in *La Mola* (0.08 ± 0.02 items/m^2^), east of the island, and *Santo Tomas* (0.08 ± 0.03 items/m^2^), south of Menorca (Table 1; Fig. 3).

**Table 2:** Results of the PERMANOVA analysis applied to test differences in sea surface microplastics within sampling period (year and month) and sampling scale (area and locality). Df, degrees of freedom; MS, mean sum of squares; pseudo-*F* value by permutation; asterisk indicates statistical significance at *P* < 0.05. *P* values based on 999 permutations

Source of variation	Sea surface MPs		
	df	MS	Pseudo-F
Year	1	0.0077	0.15
Month	2	0.0019	1.68
Area	3	0.042	4.02*
Locality	16	0.020	2.08*
Total	89

**Fig. 4:**
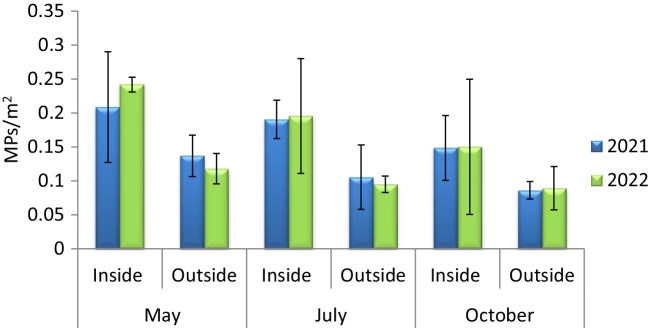
Sea surface microplastic (MP) abundances (mean ± SD) inside and outside the *Port de Maó* for both sampling years (2021 and 2022)

Given the characteristics of the east of Menorca and that two sampling sites were considered here: inside and outside the *Port de Maó*, this area was analysed separately. Results from this specific area showed that highest and significant values were obtained inside the *Port de Maó* (0.19 ± 0.02 items/m^2^; Table 3; PERMANOVA < 0.05) rather than outside (0.10 ± 0.01 items/m^2^) but no significant differences were observed according to years and months (Table 3; *p* > 0.05; PERMANOVA). In both 2021 and 2022, highest mean plastic values were observed in May and inside the *Port de Maó* (0.21 ± 0.08 items/m^2^ and 0.24 ± 0.01 items/m^2^, respectively) and lowest mean abundances were observed in October (0.09 ± 0.01 items/m^2^ and 0.09 ± 0.03items/m^2^, respectively) (Fig. 4).

**Table 3:** Results of the PERMANOVA analysis applied to test differences in sea surface microplastics within sampling period (year and month) and sampling site (inside/outside) for the *Port de Maó*. Df, degrees of freedom; MS, mean sum of squares; pseudo-*F* value by permutation; asterisk indicates statistical significance at *P* < 0.05. *P* values based on 999 permutations

Source of variation	Sea surface MPs		
	df	MS	Pseudo-F
Year	1	0.000056	0.0062
Month	2	0.0084	1.91
Site	1	0.059	8.02*
Total	35

### Shape, colour, and polymer

**Fig. 5:**
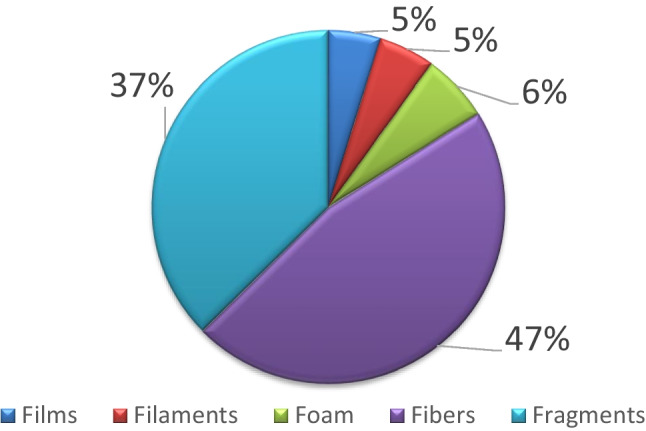
Percentage occurrence (%) of microplastic type according to identified shapes throughout the sampling area

According to shape of the identified items, fibres were the most predominant type (46%) followed by fragments (37%) throughout the sampling period and area (Fig. 5). The other shapes of items were accounted for less than 10% of the total abundance of microplastics: films (5%), pellets (0.33%), and foam (6%) (Fig. 5). It is important to highlight that during all sampling months, fibres were always predominant over fragments except in July where fibres accounted for 18% of the occurrence in 2021 and 27% in 2022, in contrast to the frequency of occurrence of fragments, 58% and 47% in 2021 and 2022, respectively. According to sampling year and areas, in 2021, for all areas except for the south, more than 40% of the items were fibres. For this year, it is outstanding that fragments in the north of Menorca accounted for 64% of the identified items and nearly 10% of the identified items in the east were foams (Table 4a). In 2022, fibres accounted for more than 40% of the identified items in all of the areas (Table 4b) and it is interesting to note that in the south, 15% of the items were films (Table 4b). During 2021 and 2022, most filaments were observed in the south of Menorca at *Macarella* (63%) and *Santo Tomas* (25%), respectively (Table 4a, b). Highest frequency of occurrence of foams was observed inside the *Port de Maó* (east) both during 2021 and 2022: 18% of occurrence in the Mussel Farm and 53% of occurrence in the Commercial Port. On the other hand, highest percentages of fibres were observed in *La Mola* (east) for both sampling years with frequency of occurrence higher than 80%. Fragments showed highest percentages in the north during 2021 (*Cala Tirant*; 78%) and along the south in 2022 (*Cala en Blanes*; 65%) (Table 4a, b). In 2021, most pellets were observed in *Cavalleria* (2%) at the north of Menorca and in 2022 at the south, *Cala Blanca* (2%) (Table 4a).

**Table 4:** Percentage occurrence (%) of microplastic type according to identified shapes according to area and locality for (a) 2021 and (b) 2022. In bold percentage of occurrence (%) according to samplig areas

a						
2021	Films	Pellets	Filaments	Foam	Fibres	Fragments
**East**	**3.93**	**0.55**	**1.79**	**9.56**	**45.70**	**38.48**
Commercial Port	12.05	0.55	1.88	6.33	23.74	55.44
Mussel Farm	7.84	0.43	3.50	17.73	38.24	32.26
Illa del Rei	0.90	1.44	0.00	17.57	53.02	27.08
La Mola	0.00	0.00	4.02	0.00	81.71	14.27
Sant Esteve	0.62	0.00	0.70	1.32	39.42	57.93
Rafalet	0.93	0.00	2.33	0.00	51.35	45.39
**North**	**5.74**	**0.37**	**3.15**	**2.02**	**24.30**	**64.42**
Port Fornells	3.93	0.00	2.37	1.78	32.30	59.62
Cavalleria	3.26	1.88	9.61	3.83	11.06	70.36
Sanitja	0.00	0.00	0.00	3.23	58.06	38.71
Cala Tirant	13.91	0.00	0.00	0.66	7.95	77.48
**South**	**0.00**	**0.33**	**17.48**	**1.84**	**43.44**	**36.90**
Cala Galdana	0.00	0.70	17.90	1.29	38.65	41.47
Macarella	0.00	0.00	62.50	0.00	0.00	37.50
Santo Tomas	0.00	0.00	57.01	0.00	0.00	42.99
Son Bou	0.00	0.00	1.78	3.20	65.30	29.72
**West**	**0.66**	**0.26**	**11.48**	**1.74**	**54.19**	**31.67**
Port Ciutadella	1.85	0.73	21.85	4.06	49.09	22.43
Cala Blanca	0.00	0.00	0.89	0.44	61.09	37.58
Cala en Blanes	0.00	0.00	9.11	0.45	54.20	36.24
b						
2022	Films	Pellets	Filaments	Foam	Fibres	Fragments
**East**	**4.26**	**0.17**	**2.47**	**19.40**	**55.94**	**17.76**
Commercial Port	1.77	0.00	4.76	52.81	33.60	7.05
Mussel Farm	9.77	0.00	1.26	15.88	48.09	25.00
Illa del Rei	5.13	0.00	3.80	8.87	57.21	24.99
La Mola	2.61	0.00	0.00	0.00	82.53	14.86
Sant Esteve	2.39	1.20	0.00	1.91	77.83	16.67
Rafalet	0.00	0.00	3.15	1.20	75.44	20.21
**North**	**6.84**	**0.39**	**3.10**	**0.20**	**48.81**	**40.67**
Port Fornells	3.91	0.91	0.00	0.46	48.90	45.81
Cavalleria	7.21	0.00	5.83	0.00	46.49	40.47
Sanitja	12.23	0.00	4.77	0.00	52.61	30.40
**South**	**14.53**	**0.27**	**9.17**	**1.13**	**43.89**	**31.00**
Cala Galdana	18.06	0.00	3.92	2.70	47.19	28.12
Macarella	15.56	0.61	5.33	0.00	38.65	39.84
Santo Tomas	7.05	0.00	24.92	1.01	49.39	17.63
**West**	**3.39**	**0.30**	**1.87**	**4.27**	**49.21**	**40.96**
Port Ciutadella	6.21	0.00	1.34	5.18	62.49	24.78
Cala Blanca	2.75	1.69	3.40	5.42	55.46	31.28
Cala en Blanes	0.36	0.00	1.77	2.66	30.64	64.57

When considering the *Port de Maó*, fibres predominate inside and outside the Port area, with values ranging from 41% (inside the Port in 2021) to 78% (outside the Port 2022). It is important to highlight that foams were the third most common category observed inside the port with a frequency of occurrence higher than 14%. Moreover, in 2022, foams appeared almost twice as much than fragments inside the port area (Table 4b).

Regarding colours of items, dark colours (black and blue) predominated in sea surface microplastics, accounting for 54.33% of the observed colours followed by light-colours, white and transparent, accounting for 16.61% and 19.36% respectively.

**Fig. 6:**
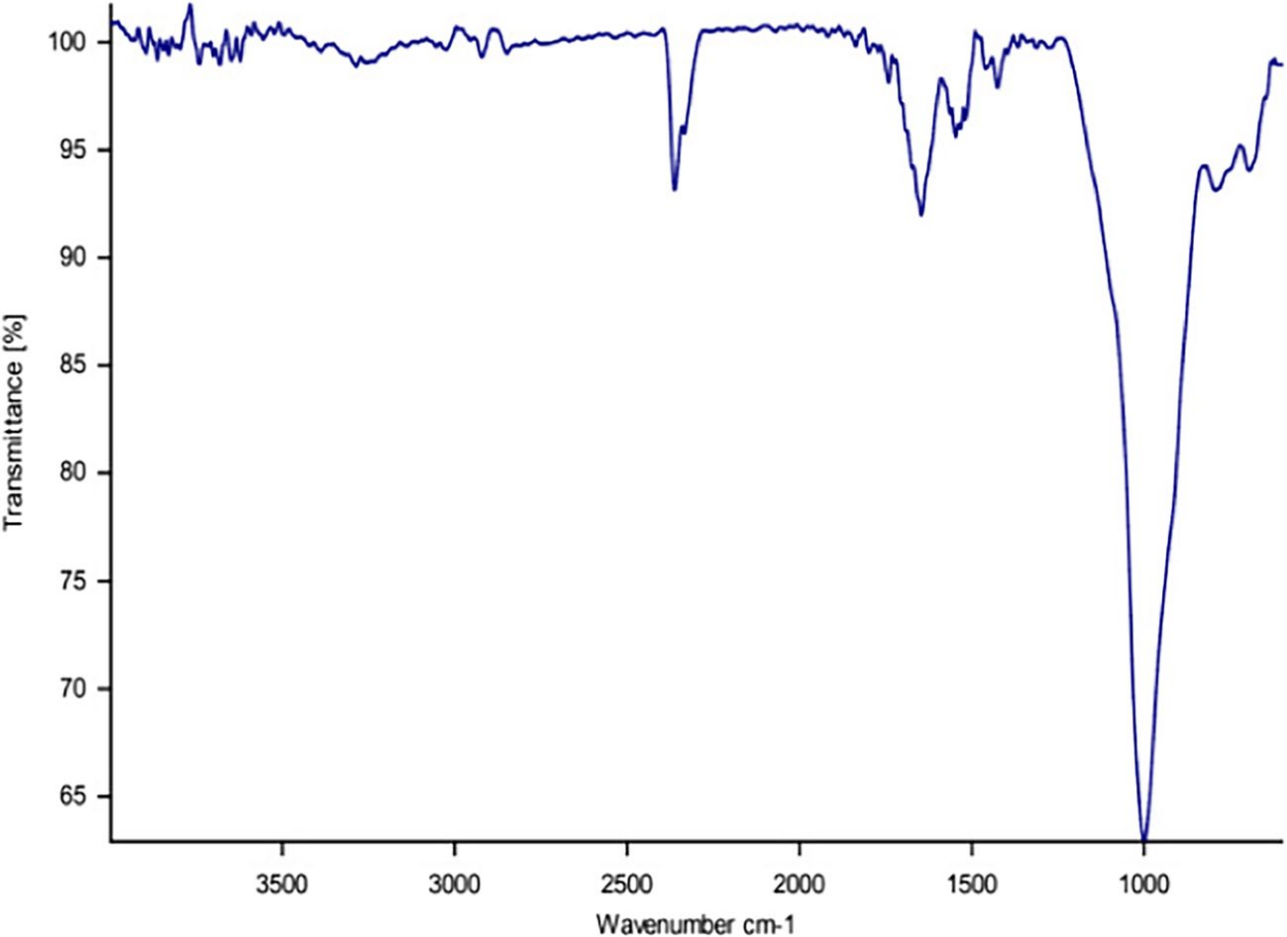
Fourier transform Infrared (FTIR) spectra (30 scans, 4000–700 cm^−1^ PerkinElmer Spectrum Spotlight 300) for expanded polystyrene (EPS) observed around the Mussel Farm in Menorca, Balearic Islands

According to the polymer characterization, microplastics quantified within the study area were made up mainly of polyethylene (PE; 48%), consisting 50% of high-density polyethylene (HDPE) and 50% of low-density polyethylene (LDPE), followed by polypropylene (PP; 13%). The third most abundant polymer was expanded polystyrene (EPS; 9%) (Fig. 6) followed by cellulose acetate (CA, 7%), styrene acrylonitrile (SAN, 7%), polycarbonate (PC, 7%), and polyvinyl alcohol (5%).

### Plastic waste management indicators

In order to explain which waste management indicators were more indicative of marine litter pollution, results from the GLM model selection indicated that the best fit model included the explanatory variables of plastic waste generated by residents population (tonnes/year/km^2^), and waste collection rate (%) (Table 5a; GLM, AIC =  − 111.0654). When exploring only the plastic fraction, results from the GLM model selection indicated that the best fit model included the explanatory variables of plastic waste generated by resident population (tonnes/year/km^2^), waste collection rate (%), and waste per capita (Table 5b; GLM, AIC =  − 119.6895).

**Table 5:** Summary of the results of the best-fit generalized linear model (GLM) for (a) all marine litter and considering plastic waste generated by residents population (Plastic residents), waste collection rate (Waste collection), and waste per capita (Waste per capita) and (b) only plastics regarding residents population (Plastic residents), waste collection rate (Waste collection), leakage tourists (Leakage tourists), and waste per capita (Waste per capita). GLM, “***” *p* < 0.001, “**” *p* < 0.01, “*” *p* < 0.05, “.” *p* < 0.1

Coefficients	Estimate	Std. error	*z* value	Pr( >|*z*|)
A				
(Intercept)	− 3.37	1.08	− 3.12	0.0025**
Plastic residents	− 0.00021	0.0000	− 2.59	0.011*
Waste collection	1.90	0.57	3.31	0.00143**
Waste per capita	0.000094	0.0000	− 1.55	0.13
B				
(Intercept)	− 6.53	2.59	− 2.53	0.014*
Plastic residents	− 0.00022	0.000079	− 2.76	0.0073**
Waste collection	3.52	1.34	2.63	0.01*
Leakage tourists	0.37	0.26	1.43	0.16
Waste per capita	0.00016	0.000076	− 2.10	0.04*

## Discussion

This study provides with further evidence of sea surface microplastics abundance in coastal areas of a small scaled island, Menorca, a UNESCO biosphere reserve. From a total of 90 samples, microplastics were observed in 100% of the samples with a mean value of 0.18 ± 0.01 items/m^2^ (180,000 ± 10,000 items/km^2^). Even though highest microplastic abundances were given in 2021 rather than in 2022, no significant differences were observed according to year or month. However significant differences were observed according to sampling area and locality suggesting that microplastic abundance is more dependent of the study area than the season of sampling.

In the absence of significant differences in microplastic abundances between years and considering the two sampling years together, highest and significant microplastic abundances were observed in the west of Menorca and the lowest abundances in the south of this island. Moreover, the highest mean microplastic abundance values in the whole study area were quantified in *Port de Ciutadella*, located in the west of Menorca, which is not surprising given that it holds a natural narrow port with maritime traffic from recreational and commercial fishing vessels and ferries (passengers and goods) which can be potentially affecting microplastic abundances in this area. Nevertheless, one could think that highest microplastic abundances should be observed in the *Port de Mao* (east of Menorca) as it is bigger and has a higher activity and capacity than the *Port de Ciutadella*. However, this is not the case and one of the reasons explaining this could be that the *Port de Ciutadella* is very narrow, acting as a natural retainer and enhancing microplastic accumulation within its waters. In this sense, in Compa et al. (2020), higher abundances of microplastics were quantified in coastal areas with a higher fractal dimension which could be the case of the *Port de Ciutadella*, as a higher fractal dimension corresponds to a higher rugosity which can facilitate the retention of plastics. Microplastic abundance quantified in the present study is in range with sea surface plastic data quantified in the Menorca Channel, specifically in the area covering from Ciutadella to the Bay of Alcudia (Mallorca), which ranges from 138,293 items/km^2^ in autumn to 347,793 items/km^2^ in spring (Ruiz-Orejón et al. 2019). Similarly, microplastics were found in all sea surface samples collected along the eastern section of the Gulf of Lion (Mediterranean Sea), and variability between samples was also observed as the highest and lowest plastic abundances were observed in the same sampling location, the Bay of Marseille (Schmidt et al. 2018). Results from this study are significantly lower than microplastics quantified in an anthropogenized area also in the Balearic Islands (Mallorca: 0.31 ± 0.09 MPs/m^2^ (Alomar et al. 2022) and also along oligotrophic Mediterranean coastal waters of Israel where a mean abundance of 7.68 ± 2.38 particles/m^3^ or 1,518,340 particles/km^2^ was observed with some areas having higher abundances of microplastics than others, but in this case, differences were not significant (van der Hal et al. 2017). Additionally, microplastic abundances where quantified along four islands in the Western Mediterranean Sea (Mallorca, Cabrera MPA, Menorca and Columbretes) and mean abundances were higher around Menorca in comparison to the other islands (Fagiano et al. 2022). However, results from the present research show that sea surface microplastic abundance in the study area is lower than values in coastal areas around Mallorca (Compa et al. 2020) and Cabrera MPA, both in the Balearic Islands and in Columbretes MPA in the western Mediterranean Sea (Fagiano et al. 2022). Variability in microplastic abundances along the Mediterranean Sea can be due to local and regional currents given that oceanographic regimes play an important role in microplastic distribution along marine ecosystems. The Balearic Islands are under the influence of the Northern Current and estimates for daily microplastic transport by this current range from 0.18 to 86.46 tonnes of microplastics reflecting the importance of nature transportation processes of this type of pollution (Schmidt et al. 2018). This natural process added to the long exposure of microplastics in the marine environment, indicated by a predominance of smaller particles in sampling stations along the Northern Current (Schmidt et al. 2018), could mean that microplastics could be finally transported to waters around the Balearic Islands including the less anthropogenized areas such as Menorca but also the more human impacted island of Mallorca. All of these factors are contributing to the variability of sea surface microplastics abundances related to source areas but also to natural processes (current transport).

Considering sampling months, highest mean values in the study area were observed in May, while lowest values were obtained in July and October which coincides with the high touristic season and post-touristic season. These results are rather surprising given that previous studies have documented highest abundances of microplastics and macroplastics during the summer season when the touristic pressure is at its highest (Compa et al. 2020). Additionally, contrary to our findings, results from a 20-month sea surface monitoring of microplastics in the North Adriatic Sea (Trieste Bay) indicated significant variability in microplastic abundances according to sampling period and this could be explained by sea surface currents affecting marine litter distribution (Gajšt et al. 2016).

According to characteristics of quantified items, fibres predominated over fragments, films, pellets, and foams which is different to previous observations in the same study area where fragments (75.35%) were the most abundant type but it has to be noted that fibres were not considered in this latter study (Fagiano et al. 2022). It is important to highlight that according to some authors, fibres are considered as one of the most concerning microplastic type given its ubiquous distribution and that they are present in species of different trophic levels (Compa et al. 2022a, b; Ois Remy et al. 2015). Moreover, in the research conducted by van der Hal et al (2017), light-coloured (white or transparent) fragments were by far more abundant than other types of plastics which also differs from our results given that dark colours (black and blue) accounted for more than 50% of the identified colours followed by light-colours (white and transparent; 36%). When considering microplastic typology, we can observe that in the *Port de Maó*, in the area with the highest commercial activity of goods, almost 50% of the identified items were foams which could be related to the transportation of packed goods through sea to this port which is higher than in the *Port de Ciutadella* where there is a higher proportion of transport of passengers in regards to goods (including material used for their packaging). On the other hand, in *Port de Ciutadella*, fibres accounted for more than 62% of the identified items which could be more indicative of terrestrial/sewage input to this area. A recent study in different layers of the marine environment (surface water, sediment, and sand) in eastern China demonstrated that highest microplastic abundance in sea surface were detected near an abandoned aquafarm followed by harbours, beaches, estuary, sewage discharge areas, operational aquafarm, and rural areas (Luo et al. 2022). Moreover, a high diversity (size, colour, shape, and type) of microplastics were observed in aquafarms, harbours, and in the recreational beach compared to other less anthropogeniezed areas indicating the complexity and diversity of pollution sources in these zones (Luo et al. 2022). In the present study, the importance of considering the natural characteristics and associated activities of the sampling area is further reinforced by the fact that when analysing microplastic abundances inside and outside the *Port de Maó* significant differences were observed with highest microplastic abundances given inside the *Port de Maó* which is an area exposed to multiple human stressors (maritime traffic, tourism, coastal urbanization) and it is also notable to indicate that a high percentage of occurrence of foams was detected here possible linked to maritime transport activities. As also reported worldwide for floating microplastics (Andrady 2011), the most abundant polymers observed in the study area were PE and PP. An unusual abundance of EPS was found, probably due to the high number of foams observed in the *Port de Ciutadella*. Cellulose acetate, making up 7% of the total microplastics characterized, is one of the most common polymers ingested by a wide range of marine organisms of different trophic levels (Fagiano et al. 2023; Ois Remy et al. 2015; Savoca et al. 2019). This polymer is released into the marine environment, mainly in the form of fibres deriving from the textile sector and cigarette butts litter (Belzagui et al. 2021; Dris et al. 2017). In the study area, a high amount of fibres have been quantified possibly link to the fact that waste water treatment plants are not yet able to retain fibres from textiles which are released to the marine environment. Cellulose acetate, when deriving from the textile sector, is generally associated with a wide range of dyes and additives that could harm marine organisms, and for this reason, it is of high ecological concern (Ois Remy et al. 2015).

Results from the model applied in the present study indicate that when considering all categories of marine litter pollution, the explanatory variables are plastic waste generated by residents population (tonnes/year/km^2^) and waste collection rate (%), whereas if only plastics are considered, the indicator regarding waste per capita (kg/hab/year) is also related to sea surface microplastic abundance along coastal areas of Menorca. It is interesting to highlight that according to the model, the indicator related to leakage from tourist population (tonnes/year) was not related to sea surface microplastics suggesting that along with the absence of temporality within microplastic abundances, sea surface plastics might be more related to everyday activities rather than to tourism and that ocean hydrodynamics possibly play a key role in sea surface microplastic distribution along coastal areas of Menorca. This idea could be reinforced by the fact that sea surface microplastics were related to waste generated per capita suggesting that a higher number of habitants in the island increases plastic loads into the marine environment, without differentiating between local people and tourists. Worldwide researchers have also concluded that in the absence of significant difference regarding the total plastic density along coastal areas, factors potentially affecting its distribution are hydrodynamics and man-made activities as unsustainable harbour operations, fisheries, and tourism (Athapaththu et al. 2020). In this line, Ripken et al (2021) pointed out that highest abundances of plastic pollution were observed in areas with human activities and that heterogeneity of microplastics were caused by several factors such as closeness to point sources (sewage outfalls, river outlets, and run-off after heavy rain fall).

Moreover, not only floating plastics should be investigated but plastic pollution from the seabed to sea surface including biota should be considered. Marine species, especially those living in coastal habitats, are at high risk of ingesting plastics (Compa et al. 2022a, b) and a recent research has documented plastic ingestion and exposure to plasticizers in fish and invertebrate (mussels and holothurians) species in a MPA of the western Mediterranean Sea (Rios-Fuster et al. 2022). Additionally, in a previous research, the overlap between seafloor plastics and microplastics ingested in demersal and benthic species was assessed suggesting that the coast off Menorca (especially outside Ciutadella) was one of the lowest impacted areas by plastic pollution in the Balearic islands (Alomar et al. 2020) and this was again observed when quantifying the risk of plastic ingestion by ichthyofauna also around the Balearic Islands and including the western area of Menorca (Compa et al. 2022a, b). These findings are in contrast with our results indicating that according to sea surface microplastics, the western area off Menorca is the most affected by plastic pollution. However, caution has to be taken when comparing results amongst different studies as different temporal and spatial scales have been considered and data has been obtained through different scientific surveys (for example coastal *vs* offshore studies), which might be influencing results on sea surface plastic distribution. In this sense, in a study covering over three decades of research regarding microplastics in plankton samples and in the digestive tracts of commercially important species (*Clupea harengus* and *Sprattus sprattus*), no changes in microplastics abundances were observed from 1987 to 2015 highlighting the need to better and further understand how plastics circulate through the marine environment (Beer et al. 2018).

Given the fact that there are estimates that approximately 1.5–4% of the global plastic production enters the ocean annually (Tsiaras et al. 2022) and that these amounts are expected to increase by 1 order of magnitude in the following years (by 2025) (Maes et al. 2021), it is difficult to assume in the following years a decrease of plastic pollution and its effects upon marine biodiversity. Once more, results from this research and previous studies reflect the importance of studying within a holistic approach the whole marine ecosystem when assessing plastic pollution (Fagiano et al. 2023) in order to understand the extent to which plastic pollution effects marine environments. Moreover, given that litter is transported across oceans and seas having the potential to produce impacts in areas far away from their source, it is very important to conduct and adopt plans to mitigate and fight against marine pollution at least at a regional scale. In the Mediterranean Sea, in 1995, the Barcelona Convention for the Protection of the Marine Environment and the Coastal Region of the Mediterranean was adopted with one of its goals being the prevention and reduction of pollution in order to protect the marine environment and coastal zones. Moreover, at European level, the Marine Strategy Framework Directive was adopted in 2008 in order to achieve a Good Environmental Status (GES) of European seas and oceans according to 11 descriptors, being marine litter one of them. The implementation of these policies requires the development and application of harmonized and standardized protocols in order to quantify and characterize litter in marine ecosystems allowing for the identification of sources and sinks and particularly areas exposed to this type of pollution. At a local level, in the Balearic Islands, the autonomous government implemented the Balearic law for waste and polluted lands (law 8/2019) in March 2019 and scientific data gathered on marine litter in the past years, like the set of data presented in this study, as well as ongoing monitoring and scientific surveys will allow to evaluate the effectiveness of this law in the short-mid-term (within 5–10 years).

## Conclusions

Results from this study reflect, once more, that sea surface microplastics are present in all sea surface water samples around coastal areas of a Mediterranean island. In addition, no significant differences were observed according to sampling period but significant differences were observed regarding sampling area (both area and locality) suggesting that sea surface plastics might be more dependent of the spatial scale rather than the temporal scale. A very short time series (2 years) has been considered in this research and recurrent sampling and analysis within a wider temporal and spatial scale is needed in order to understand plastic pollution dynamics in marine ecosystems. With this research we provided data regarding sea surface microplastics obtained through a harmonized protocol from an established monitoring program which will be conducted over the years making feasible the gathering of reliable data within a temporal scale. Moreover, this will facilitate assessing the evolution of microplastic pollution in coastal areas at a spatial and temporal scale as well as providing with new reliable data to be used in the definition of baseline and threshold values and evaluation of GES.

## References

[CR1] Acosta J, Canals M, Carbó A, Muñoz A, Urgeles R, Muñoz-Martín A, Uchupi E (2004). Sea floor morphology and plio-quaternary sedimentary cover of the Mallorca channel, Balearic islands, western Mediterranean. Mar Geol.

[CR2] Alomar C, Estarellas F, Deudero S (2016). Microplastics in the Mediterranean sea: deposition in coastal shallow sediments, spatial variation and preferential grain size. Mar Environ Res.

[CR3] Alomar C, Deudero S, Compa M, Guijarro B (2020). Exploring the relation between plastic ingestion in species and its presence in seafloor bottoms. Mar Pollut Bull.

[CR4] Alomar C, Compa M, Sanz-Martin M, Fagiano V, Álvarez E, Valencia JM, Deudero S (2022). A holistic approach to plastic pollution in integrated multi-trophic aquaculture facilities: plastic ingestion in Sparus aurata and *Mytilus gallopr*ovincialis. Aquaculture.

[CR5] Alomar C (2020) Plastic litter in seafloor habitats of the Balearic Islands and its implications for marine species. PhD thesis. Universitat de les Illes Balears, 202pp

[CR6] Anderson MJ, Gorley RN, Clarke KR (2008). PERMANOVA+ for PRIMER: guide to software and statistical methods.

[CR7] Andrady AL (2011). Microplastics in the marine environment. Mar Pollut Bull.

[CR8] Athapaththu AMAIK, Thushari GGN, Dias PCB, Abeygunawardena AP, Egodauyana KPUT, Liyanage NPP, ... Senevirathna JDM (2020) Plastics in surface water of southern coastal belt of Sri Lanka (Northern Indian Ocean): distribution and characterization by FTIR. Mar Pollut Bull 161:111750 10.1016/j.marpolbul.2020.111750 33132148

[CR9] Beer S, Garm A, Huwer B, Dierking J, Nielsen TG (2018). No increase in marine microplastic concentration over the last three decades–a case study from the Baltic Sea. Sci Total Environ.

[CR10] Belzagui F, Buscio V, Gutiérrez-Bouzán C, Vilaseca M (2021). Cigarette butts as a microfiber source with a microplastic level of concern. Sci Total Environ.

[CR11] Boucher J, Bilard G (2020). The Mediterranean: Mare plasticum.

[CR12] Cohen-Sánchez A, Solomando A, Pinya S, Tejada S, Valencia JM, Box A, Sureda A (2022). First detection of microplastics in Xyrichtys novacula (Linnaeus 1758) digestive tract from Eivissa Island (Western Mediterranean). Environ Sci Pollut Res.

[CR13] Compa M, Alomar C, Mourre B, March D, Tintoré J, Deudero S (2020). Nearshore spatio-temporal sea surface trawls of plastic debris in the Balearic Islands. Mar Environ Res.

[CR14] Compa M, Alomar C, López Cortès MF, Rios-Fuster B, Morató M, Capó X, Fagiano V, Deudero S (2022). Multispecies assessment of anthropogenic particle ingestion in a marine protected area. Biology.

[CR15] Compa M, Wilcox C, Hardesty BD, Alomar C, March D, Deudero S (2022b) Quantifying the risk of plastic ingestion by ichthyofauna in the Balearic Islands (western Mediterranean Sea). Mar Pollut Bull 183:114075 10.1016/j.marpolbul.2022.114075 36084610

[CR16] Dris R, Gasperi J, Mirande C, Mandin C, Guerrouache M, Langlois V, Tassin B (2017). A first overview of textile fibers, including microplastics, in indoor and outdoor environments. Environ Pollut.

[CR17] Fagiano V, Compa M, Alomar C, García-Marcos K, Deudero S (2022). Marine plastics in Mediterranean islands: evaluating the distribution and composition of plastic pollution in the surface waters along four islands of the Western Sea Basin. Environ Pollut.

[CR18] Fagiano V, Compa M, Alomar C, Rios-Fuster B, Morató M, Capó X, Deudero S (2023). Breaking the paradigm: Marine sediments hold two-fold microplastics than sea surface waters and are dominated by fibers. Sci Total Environ.

[CR19] Gajšt T, Bizjak T, Palatinus A, Liubartseva S, Kržan A (2016). Sea surface microplastics in Slovenian part of the Northern Adriatic. Mar Pollut Bull.

[CR20] Galgani F, Hanke G, Werner S, Oosterbaan L, Nilsson P, Fleet D, Kinsey S, Thompson RC, van Franeker J, Vlachogianni T, Scoullos M, Veiga JM, Palatinus A, Matiddi M, Maes T, Korpinen S, Budziak A, Leslie H, Gago J, Liebezeit G (2013) Guidance on monitoring for marine litter in European seas. MSFD Technical Subgroup on Marine Litter (TSG-ML). European Commission. Joint Research Centre Scientific and Policy, Institute for Environment and Sustainability Reports 2013

[CR21] Hatzonikolakis Y, Giakoumi S, Raitsos DE, Tsiaras K, Kalaroni S, Triantaphyllidis G, Triantafyllou G (2022). Quantifying transboundary plastic pollution in marine protected areas across the Mediterranean Sea. Front Mar Sci.

[CR22] IUCN-EA-QUANTIS (2020) National Guidance for plastic pollution hotspotting and shaping action, Country report Menorca

[CR23] Löder MGJ, Kuczera M, Mintenig S, Lorenz C, Gerdts G (2015). Focal plane array detector-based micro-Fourier-transform infrared imaging for the analysis of microplastics in environmental samples. Environ Chem.

[CR24] Luo Y, Sun C, Li C, Liu Y, Zhao S, Li Y, ..., Li F (2022) Spatial Patterns of Microplastics in Surface Seawater, Sediment, and Sand Along Qingdao Coastal Environment. Front Mar Sci 9:916859

[CR25] Macias D, Cózar A, Garcia-Gorriz E, González-Fernández D, Stips A (2019). Surface water circulation develops seasonally changing patterns of floating litter accumulation in the Mediterranean Sea. A modelling approach. Mar Pollut Bull.

[CR26] Maes T, McGlade J, Fahim IS, Green DS, Landrigan P, Andrady AL, ... Turra A (2021) From pollution to solution: a global assessment of marine litter and plastic pollution. United Nations Environment Programme

[CR27] Ois Remy F, Collard F, Gilbert B, Compè P, Eppe G, Lepoint G (2015) When microplastic is not plastic: the ingestion of artificial cellulose fibers by macrofauna living in seagrass macrophytodetritus. 10.1021/acs.est.5b02005 10.1021/acs.est.5b02005 26301775

[CR28] Rios-Fuster B, Alomar C, González GP, Martínez RMG, Rojas DLS, Hernando PF, Deudero S (2022). Assessing microplastic ingestion and occurrence of bisphenols and phthalates in bivalves, fish and holothurians from a mediterranean marine protected area. Environ Res.

[CR29] Rios-Fuster B, Compa M, Alomar C, Morató M, Ryfer D, Villalonga M, Deudero S (2023). Are seafloor habitats influencing the distribution of microplastics in coastal sediments of a Marine Protected Area?. Environ Sci Pollut Res.

[CR30] Ripken C, Kotsifaki DG, Chormaic SN (2021). Analysis of small microplastics in coastal surface water samples of the subtropical island of Okinawa, Japan. Sci Total Environ.

[CR31] Ripley B (2011). MASS: support functions and datasets for Venables and Ripley’s MASS. R package version.

[CR32] Ruiz-Orejón LF, Mourre B, Sardá R, Tintoré J, Ramis-Pujol J (2019). Quarterly variability of floating plastic debris in the marine protected area of the Menorca Channel (Spain). Environ Pollut.

[CR33] Savoca S, Capillo G, Mancuso M, Faggio C, Panarello G, Crupi R, Bonsignore M, D’Urso L, Compagnini G, Neri F, Fazio E, Romeo T, Bottari T, Spanò N (2019). Detection of artificial cellulose microfibers in Boops boops from the northern coasts of Sicily (Central Mediterranean). Sci Total Environ.

[CR34] Schmidt N, Thibault D, Galgani F, Paluselli A, Sempéré R (2018). Occurrence of microplastics in surface waters of the Gulf of Lion (NW Mediterranean Sea). Prog Oceanogr.

[CR35] Suaria G, Avio CG, Mineo A, Lattin GL, Magaldi MG, Belmonte G, Moore CJ, Regoli F, Aliani S (2016). The Mediterranean plastic soup: synthetic polymers in Mediterranean surface waters. Sci Rep.

[CR36] Tesán Onrubia JA, Djaoudi K, Borgogno F, Canuto S, Angeletti B, Besio G, ... Lenoble V (2021) Quantification of microplastics in north-western Mediterranean harbors: seasonality and biofilm-related metallic contaminants. J Mar Sci Eng 9(3):337

[CR37] Tsiaras K, Costa E, Morgana S, Gambardella C, Piazza V, Faimali M, ... Garaventa F (2022) Microplastics in the Mediterranean: variability from observations and model analysis. Front Mar Sci 9:288

[CR38] Van der Hal N, Ariel A, Angel DL (2017). Exceptionally high abundances of microplastics in the oligotrophic Israeli Mediterranean coastal waters. Mar Pollut Bull.

